# 3D Nanoprinting via laser-assisted electron beam induced deposition: growth kinetics, enhanced purity, and electrical resistivity

**DOI:** 10.3762/bjnano.8.83

**Published:** 2017-04-07

**Authors:** Brett B Lewis, Robert Winkler, Xiahan Sang, Pushpa R Pudasaini, Michael G Stanford, Harald Plank, Raymond R Unocic, Jason D Fowlkes, Philip D Rack

**Affiliations:** 1 Materials Science and Engineering Department, University of Tennessee, Knoxville, TN 37996, USA; 2 Graz Centre for Electron Microscopy, Steyrergasse 17, 8010 Graz, Austria; 3 Center for Nanophase Materials Sciences, Oak Ridge National Laboratory, Oak Ridge, TN 37381, USA; 4 Institute of Electron Microscopy and Nanoanalysis, Graz University of Technology, Steyrergasse 17, 8010 Graz, Austria

**Keywords:** additive manufacturing, beam induced processing, 3D printing, direct-write, electron beam induced deposition, microscopy, nanofabrication, pulsed laser, purification, rapid prototyping

## Abstract

We investigate the growth, purity, grain structure/morphology, and electrical resistivity of 3D platinum nanowires synthesized via electron beam induced deposition with and without an in situ pulsed laser assist process which photothermally couples to the growing Pt–C deposits. Notably, we demonstrate: 1) higher platinum concentration and a coalescence of the otherwise Pt–C nanogranular material, 2) a slight enhancement in the deposit resolution and 3) a 100-fold improvement in the conductivity of suspended nanowires grown with the in situ photothermal assist process, while retaining a high degree of shape fidelity.

## Introduction

The first fully incorporated 3D transistor logic was reported in 2012 [1]. Further 3D device concepts and architectures will require the development of new 3D nanoscale fabrication techniques which will inevitably enhance performance and add functionality to nanoscale devices. Emerging applications include, but are not limited to, high strength nanolattices [2], optical metamaterials [3], accurate molecular detection [4], the study of biological systems important in determining cancer treatment options [5], and reliable, low cost, high performance magnetic hard disk drives [6]. A variety of fabrication techniques have been used to construct multi-dimensional nanostructures [7–10] with differing degrees of success.

Recently, electron beam induced deposition (EBID) was extended to 3D nanoscale mesh geometries [11]. Deposition occurs during EBID as the nanoscale focused electron beam dissociates adsorbed precursor molecules. A condensed byproduct accumulates by prolonged electron exposure with the shape and composition of the resulting deposit dictated by both the electron beam scanning parameters and the properties of the precursor. The resolution/size of the deposit is determined by the electron probe size and the interaction between the electron beam, substrate, and dynamic growth front which generates subsequent back-scattered (BSE), forward-scattered (FSE), and secondary electrons (SE). Conveniently, EBID has the advantage of being compatible with a wide range of precursor and substrate materials [12]. Several applications have been explored with EBID and focused electron beam induced etching including: sensors [13–15], field emission cathodes [16–17], plasmonic elements [3,18], lithographic mask repair [19–21], scanning probe tips [22–25], photonic materials [26], magnetic materials [27–28], nanoparticle separations [29], and lithographic techniques [30–31] to name a few. While standard patterning of the electron beam has resulted in complex 2D deposits of arbitrary shape, care must be taken as subtle proximity effects can be minimized or exacerbated in some electron beam [32], gas flux and patterning [33], and temperature regimes [34]. Moreover, while several examples of 3D growth have been demonstrated [35–38] beyond simple 1D nanowires, controlled growth of complex geometries using EBID has only recently been achieved based on a combined simulation and computer aided design approach [11]. This approach has also been used with Ga^+^ ion beam induced deposition (IBID) with a great degree of success, albeit at slightly larger dimensions [39].

A well-known disadvantage of EBID is the incorporation of carbonaceous byproducts due to the use of organometallic precursors with relatively large carbon atomic fractions. The resulting deposit typically consists of metallic nanocrystals suspended in a non-metallic (often carbonaceous) matrix. There are, however, a few documented examples of precursors that produce relatively pure deposits without additional purification steps [40–43], and there has been a recent thrust to design and evaluate precursors tailor-made for electron stimulated reactions [44–45], but options for obtaining pure EBID nanostructures are still very limited.

More immediately promising, various methods have been developed to purify EBID deposits. For instance, those which involve post-growth electron-stimulated impurity removal with and without a co-reactant (ex situ) [46–49] and/or facilitated by a reactive gas co-flow during deposition (in situ) [50–52]. A third purification method employs thermal energy either by substrate heating [53] or locally heating the substrate using a pulsed laser system [54–56]. For 3D objects, in situ purification methods are critical in applications requiring high fidelity shape retention due to the significant volumetric contraction associated with impurity removal [57].

In situ purification of complex 3D nanostructures is a significant challenge due to the large parameter space and multiple interactions that are involved. The removal of impurities during the purification process competes with the simultaneous deposition of the desired material. Additionally, the deposition rate is related to the surface coverage of the precursor on the substrate [33] and therefore an additional co-reactant can compete for surface sites changing the growth rate [58]. Higher temperatures can reduce the precursor residence time further complicating the growth rate [34].

In a previous work [54], we introduced a method for using a pulsed laser system to purify in situ EBID deposits. That work dealt with the deposition of 2D pads and a simple pillar structure. The primary thrust of this current work illuminates the non-trivial aspects of transferring the laser-assisted purification process to the complex 3D growth. Specifically, the coupled relationship between vertical and lateral growth is an important consideration, making the accurate construction of complex structures very difficult. Additionally, this work represents a follow up to the analysis of the morphological structure of carbon as a function of laser irradiance, showing how graphitization can have an effect on the extent of purification. Furthermore, no deliberate synchronization was employed between the EBID and laser assist as we have determined that the low laser duty cycle minimally depletes the growth during the simultaneous electron and laser on-times. This allows for the duty cycle of the laser to be uncoupled from the electron beam dwell time while at the same time maintaining minimal thermal drift. Finally, in this work, we investigate the effect of reactive gas on the laser-assisted in situ purification of 3D features synthesized using EBID from the commonly employed precursor MeCpPt(IV)Me_3_. Notably, we explore the critical electron, precursor, and laser parameters necessary to maintain high fidelity while simultaneously promoting high purity and low electrical resistivity.

## Experimental

### Electron beam induced deposition

Platinum nanostructures were grown onto a silicon substrate from the MeCpPt(IV)Me_3_ precursor gas using an FEI NOVA 600 dual-beam system equipped with multiple gas injection systems (GIS). Before loading the Si substrate, it was cleaned via sonication in acetone for 5 minutes and rinsed in isopropanol before drying. The precursor gas was injected using a FEI GIS and the temperature was held at 45 °C for the standard conditions and varied as discussed to control the precursor flux. 3D structures were patterned by controlling the spatial coordinates of the electron beam as well as the dwell time at each point via a text file read by the microscope software. For all patterns, a pixel point pitch of 1 nm was used while varying dwell times per pixel. A previously developed computer aided design program was used to determine the dwell times per pixel necessary to construct complex shapes. The beam energy and current for all patterns was set at 30 keV and 21 pA, respectively.

### Laser delivery system

A 915 nm wavelength 25 W multichip diode laser made by Oclaro Inc. was used as the source for laser irradiation. A PCX-7410 laser diode driver (DEI^®^) was used to control the pulse width, frequency, and power. The particular parameters were chosen based on initial experiments that resulted in heating sufficient to initiate carbon removal without inducing significant thermal drift and/or laser chemical vapor deposition (CVD). Laser pulses are delivered to the sample with an optical working distance of 9 mm using a multi-mode 100 µm diameter fiber optic cable housed within a stainless steel shaft with automated axial translation. An objective lens located at the shaft tip projects the 100 µm diameter laser spot with a Gaussian distribution at the coincidence point between the focused electron beam and the gas injection systems. The laser delivery system is a prototype under development by Waviks, Inc. This system was mounted on an FEI Novalab 600 chamber port oriented at 52° with respect to the silicon substrate plane. Additional information on the laser system can be found in [59].

### Gas injection system

An FEI gas injector was used to deliver the MeCpPt(IV)Me_3_ precursor close to the substrate surface. The bottom of the gas nozzle was located 100 µm above the silicon surface. In the substrate plane, the nozzle was located 250 µm from the beam impact point; this distance was measured from the top of the nozzle provided in a secondary electron image acquired at normal incidence with respect to the substrate. A uniform chamber pressure of ≈1 × 10^−5^ mbar was established prior to EBID by continuous precursor flow. The base chamber pressure of the microscope is roughly 2 × 10^−6^ mbar.

**Figure 1:**
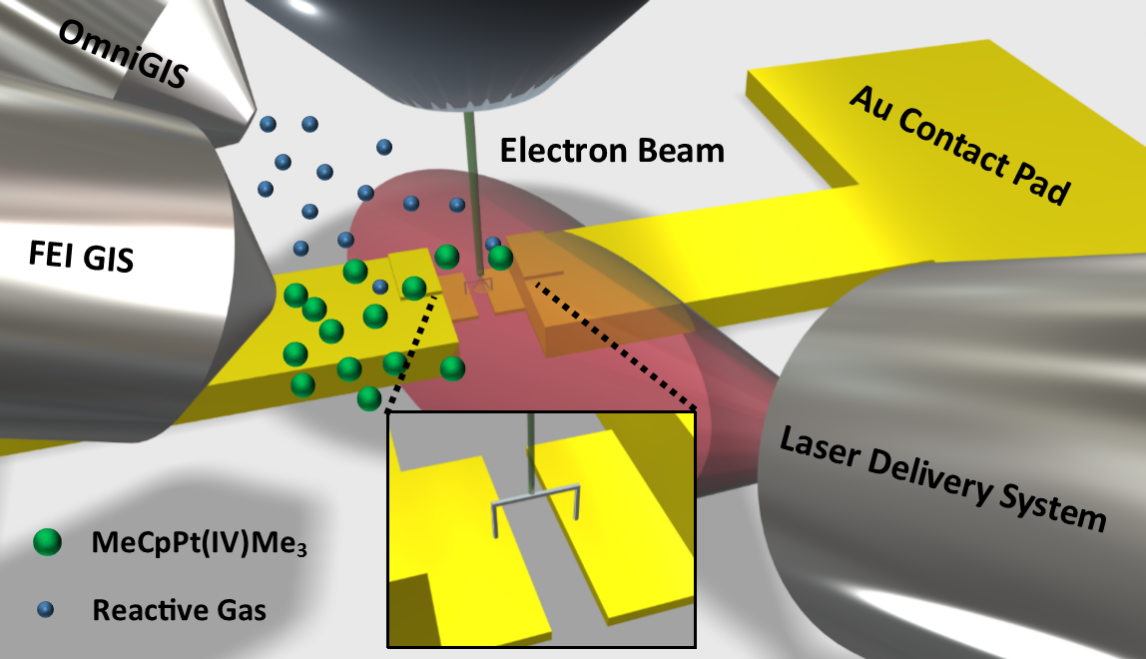
Schematic illustrating the experimental system which includes a laser delivery system, precursor and co-reactant gas delivery systems, and the electron beam all coincident to the same region. The schematic also illustrates the deposition of the 3D suspended bridge structures grown for the electrical measurements.

An OmniGIS I was used to introduce a reactive gas co-flow of Ar–O_2_ (80–20 atom %) to the system during deposition. The gas nozzle was located 150 µm above the silicon surface and 350 µm laterally and the chamber pressure during co-flow was ≈1.8 × 10^−5^ mbar. The temperature of the Ar–O_2_ gas was room temperature (23 °C) and the OmniGIS I was mounted on a separate high angle (52°) port on the SEM chamber located roughly 45° relative to the FEI GIS. Figure 1 is a schematic illustrating the geometry of the gas and laser delivery systems relative to the substrate and electron beam impact point.

### STEM imaging and EELS analysis

Scanning transmission electron microscope (STEM) imaging and electron energy loss spectroscopy (EELS) were performed using a Nion UltraSTEM 100 which is equipped with aberration correction of the probe forming lens. Beam-induced damage and contamination were minimized by using an accelerating voltage of 60 kV and a 40 pA beam current. High angle annular dark field (HAADF) and bright field (BF) STEM imaging was used to analyze the structure of the nanoscale deposits before and after laser annealing. EELS was performed in order to determine the structure of carbon through analysis of the carbon K-edge.

### Electrical device fabrication and measurements

A two-contact electrical test structure was created using conventional nanofabrication methods in order to measure the electrical resistivity of freestanding nanoscale bridges. Geometric constraints (the freestanding nature of the nanobridge) necessitated a two-point probe configuration over the standard four-point measurement. A combination of photolithography and electron beam lithography (EBL) were used to produce the two-contact pads with a spacing of 500 nm. An initial set of gold electrical contacts were patterned using photolithography and deposited with a thickness of 100 nm. A 3 nm titanium adhesion layer was deposited, prior to gold sputtering, to promote adhesion between the gold contacts and the underlying, insulating SiO_2_ film (290 nm). The original photolithographically defined spacing between electrical contacts was 20 µm. EBL was performed using a Raith ELPHY Quantum patterning engine equipped on the FEI NovaLab 600 dual beam. The contact spacing was reduced to 500 nm using EBL. The EBL contact extensions were 20 nm thick gold with a 3 nm titanium adhesion layer. After patterning, the substrate was cleaned via sonication for 5 minutes in an acetone bath. EBID was then used to construct a 3D bridge across the 500 nm gap. Figure 1 shows a schematic of the location of the bridge in relation to the contact pads.

Electrical measurements were made using an Agilent Technologies B1500 semiconductor parameter analyzer in a two-point probe configuration. The source voltage was swept from −1 to 1 V in 0.01 V increments and the current was measured as a function of voltage. Wire resistance was taken as the slope of the best linear fit of the *I*–*V* curve. High resolution SEM images of each wire were used to estimate the length and cross-sectional area to calculate the resistivity of each wire.

## Results and Discussion

### Growth rates

**Figure 2:**
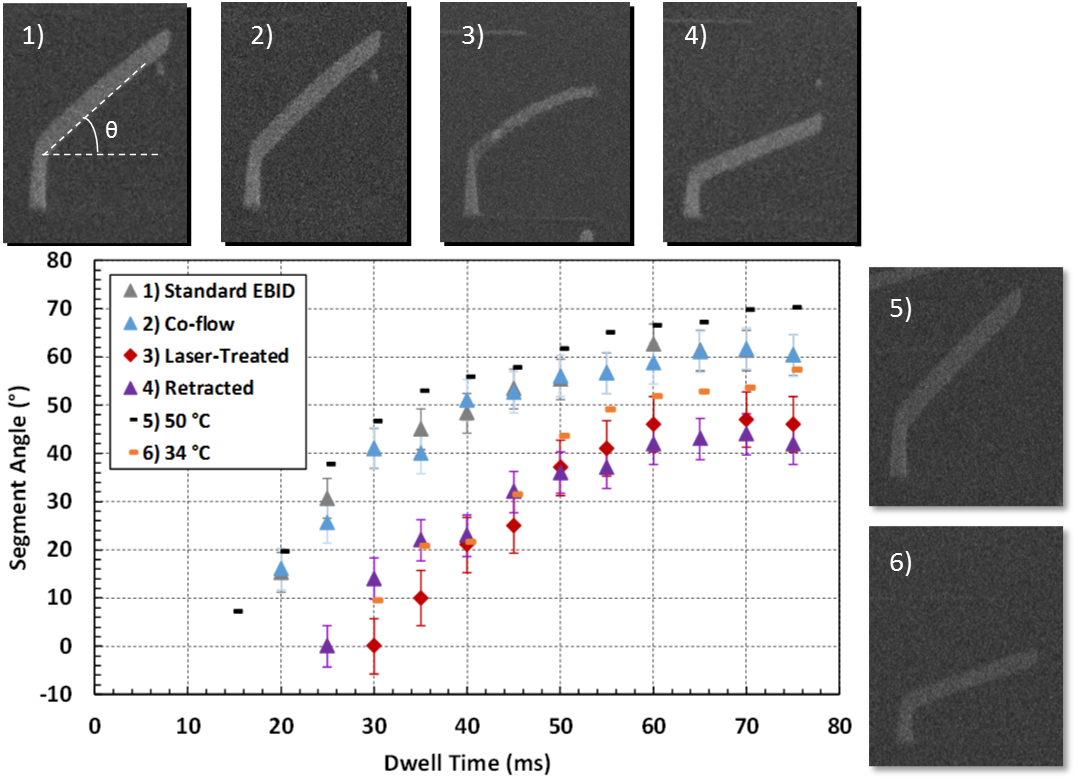
Segment angle plotted as a function of dwell time per pixel grown under six different conditions: 1) EBID with standard precursor temperature at 45 °C and a resultant chamber pressure of 1.2 × 10^−5^ mbar (gray). 2) EBID standard Pt precursor conditions with an argon-oxygen co-flow at 1.83 × 10^−5^ mbar (blue). 3) EBID with standard Pt precursor conditions with laser irradiation at a pulse width and frequency of 10 µs and 100 Hz, respectively (red). 4) EBID with standard Pt precursor conditions with a retracted nozzle and lower precursor flux (purple). 5) and 6) EBID with the precursor temperature raised or lowered, respectively, as indicated (black and orange). SEM images of the resulting pillar and segment, for each growth condition, are provided, where a common dwell time of 40 ms was used for each EBID condition. The SEM images were acquired at a tilt angle of 52° with respect to the plane containing the pillar and segment. For scale, the projection of each cantilever arm is 400 nm.

The relation between the vertical and lateral growth rates is a critical parameter required to accurately and reproducibly construct 3D nanostructures. Once this relation is known, the beam dwell time and pitch can be adjusted as necessary to construct more complex shapes [60]. A simple unit demonstrating this relationship is the angle between a grown vertical post and a cantilever arm, depicted as θ in Figure 2. This angle will be termed “segment angle” hereafter. The vertical pillar is grown by parking the electron beam at a particular spot for 8 seconds, and the segment is grown off the pillar by stepping the electron beam in 1 nm increments for a given dwell time per point. The segment angle was determined for the following EBID conditions: 1) standard EBID (45 °C precursor and standard precursor nozzle position), 2) EBID with co-reactant Ar–O_2_ flow, 3) EBID with in situ laser assist, 4) EBID with a retracted nozzle, and EBID with 5) higher and 6) lower gas flux as modified by the precursor temperature (34 °C and 50 °C). Carbon content and growth rate is strongly affected by the particular EBID condition used.

The best purity that can be achieved using an optimized beam voltage and current during EBID is PtC_5_ [61]. Ex situ thermal annealing has been used to remove carbon in the past [47] but causes severe structural distortions due to the large quantity of carbon removed. Thus, in situ purification is necessary for all but the simplest geometries because of severe volumetric reduction (see Supporting Information File 1 for 3D examples).

Pulsed laser irradiation and gas pressure both affect the growth rate and morphology of the EBID structures. A useful way to characterize this change is by fabricating a pillar + cantilever, or ‘segment’, using EBID. Figure 2 depicts the changes in segment angle versus dwell time for several different growth conditions.

The co-reactant flow (Figure 2, case #2) EBID studied here affects neither growth rate nor segment angle, relative to standard EBID (Figure 2, case #1). Relatively weak O_2_–C binding is thought to govern this behavior even though the localized partial pressure of the co-reactant oxygen gas is estimated to be on the order of 1.5× higher. The O_2_–C residence time is short (20 ns [62]) relative to the MeCpPt(IV)Me_3_ precursor residence time (reported values ranging from 29 µs [63] to 30 ms [64]). The result is a negligible O_2_ surface coverage which inhibits the purification reaction. As will be demonstrated below, the resultant 3D composition at these lower beam currents is not appreciably affected, contrary to growth conditions at higher beam currents and much higher partial pressures [50].

Laser-assisted EBID (LAEBID) clearly impacts the final deposit growth and morphology (Figure 2, case #3). In this case, the segment angle decreases reflecting a decrease in the vertical growth rate per pixel dwell. The reduced growth rate is a result of two contributing factors, namely: 1) densification and carbon reduction [54] and 2) reduced average precursor coverage due to the periodic laser heating. Segment volume per unit length is clearly less for the laser-assist case. Comparing the segment grown with and without the laser-assist, it is clear that the width of the segment with the laser-assist is narrower, indicative of a reduced growth rate and densification (discussed in more detail below).

The effective precursor surface coverage was decreased using two different EBID configurations. In one case (#4), the precursor delivery nozzle was retracted and in another (#6) the precursor reservoir temperature was lowered from 45 to 34 °C. Both approaches yield a comparable segment angle to the laser-assisted EBID – thus it is opined that the photothermal heating must produce an equilibrium precursor surface coverage comparable with the lower precursor flux growth. As expected, the higher Pt precursor temperature (#5) has a higher growth rate and higher segment angle for comparable dwell times.

### STEM imaging and EELS

**Figure 3:**
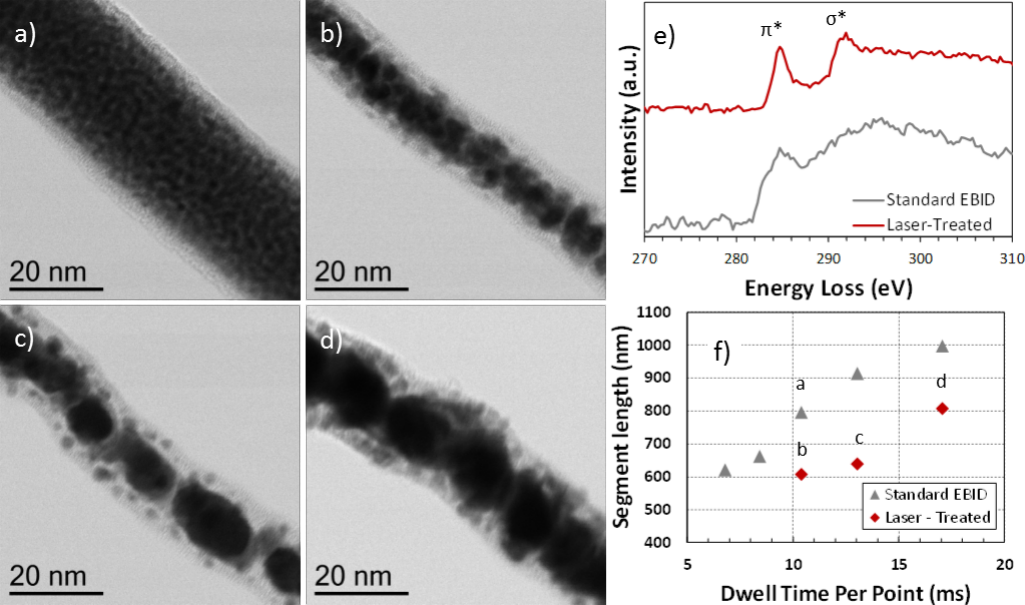
BF STEM images of a) an as-deposited EBID segment with a 10.4 ms dwell time per pixel and in situ LAEBID performed at various dwell times per point including b) 10.4 ms, c) 13.0 ms and d) 17.0 ms. e) EELS spectra obtained from as-deposited and laser-treated segments grown with a dwell time of 17 ms per point. f) Plot of segment length as a function of dwell time for both standard EBID segments and laser-treated EBID segments. The alphabetic labels highlight the points corresponding to the BF STEM images in parts a)–d).

STEM was used to investigate the morphology of the as-deposited and laser treated structures. In addition, the chemical nature of the carbon was characterized for different EBID configurations using EELS. Figure 3 provides insight into the purification mechanisms during LAEBID. Figure 3a shows a high resolution STEM image of a standard EBID segment grown off a silicon wafer edge in order to avoid substrate interactions during beam transmission. The electron beam dwell time per pixel was 10.4 ms (see Supporting Information File 1, Figure S5 for HAADF STEM images of the standard EBID deposits). In total, five segments extending 600 nm in lateral displacement beyond the wafer edge were grown using dwell times: 6.78, 8.44, 10.4, 13.0, and 17.0 ms per point. The associated take-off angles for these deposits were 15°, 25°, 41°, 49°, and 53°, respectively. Note that the dwell times here are shorter because the precursor source was re-filled after running the calibration curves in section ‘Growth rates’. Five segments were also grown with the same dwell time range while simultaneously irradiating with the laser at a pulse width of 500 ns and a frequency of 20 Hz. The gentler laser conditions were necessary because the edge of the wafer acted as a thermal boundary, effectively increasing the rate of heating as compared to structures grown on a bulk substrate. Figure 3b–d shows the segments corresponding to dwell times of 10.4, 13.0, and 17.0 ms per point, respectively, with take-off angles 9°, 20°, and 42°, respectively. The lower dwell time segments had growth rates that were insufficiently high for continuous lateral growth, therefore “falling off” the edge.

Figure 3a–d shows the stark difference between as-deposited EBID structures and the laser treated structures. Here, Pt-rich regions are the relatively dark regions in the BF STEM images. Most notably, laser exposure induces platinum particle consolidation and growth while simultaneously driving a reduction in segment volume associated with the removal of carbon as well as carbon densification/graphitization. Figure 3f shows a plot of the segment lengths as a function of dwell time per point for both the standard EBID and the laser-assisted EBID structures. The change in length is a byproduct of the change in angle that occurs from larger vertical growth and constant lateral growth. In addition to the change in length, both the EBID and LAEBID segment widths increase as a function of increasing dwell time, which is consistent with standard EBID and IBID growth models [65]. Temperature-dependent diffusion drives grain growth favoring a reduction in platinum surface area [66–67]. Comparing Figure 3b–d, it is clear that the longer dwell time segments experience more grain coarsening and thus must realize a higher temperature. While higher absorption for the wider deposits grown at longer dwell time was initially suspected to cause the increased grain coarsening, thermal simulations (see below) reveal that the longer total length of the pillars at higher dwell times is the dominant factor.

EELS was performed to determine the influence of the laser annealing treatment on the evolution of the carbon structure contained within the deposited pillar. The carbon K-edge provides information on the electronic structure of carbon and analysis of characteristic features contained within the energy loss near edge structure (ELNES) indicates whether carbon is amorphous, graphitic or diamond. Figure 3e shows the corresponding background subtracted carbon K-edge EEL spectra from the as-deposited (black) pillar and the laser-treated (red) pillar. Each spectra contains the π* and σ* that peaks at ≈285 eV and ≈291.5 eV, respectively. The carbon K-edge from the as-deposited samples shows diffuse π* peak and σ* peak which closely resemble that of amorphous carbon [68]. Following the laser treatment, the characteristic π* and σ* peaks have sharper spectral features, indicating that the carbon has been transformed from amorphous to graphitic. The laser treatment thus induces C phase transformation from amorphous to graphite, which is consistent with other higher temperature studies [69–70] involving EBID carbon.

### Electrical measurements

**Figure 4:**
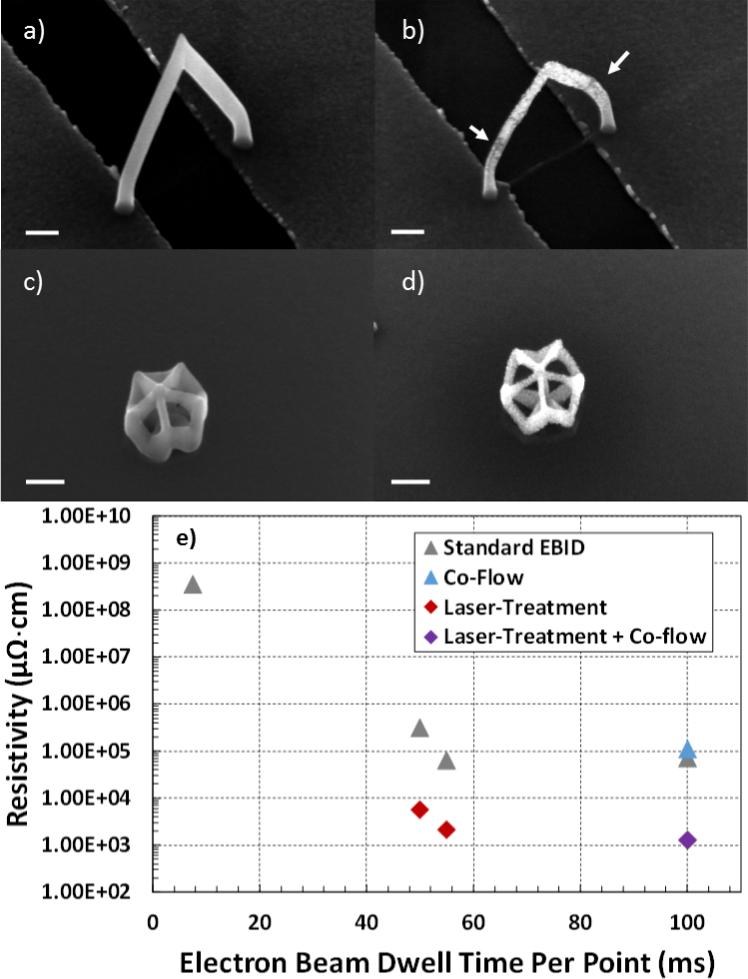
SEM images (scale bar length is 100 nm) of suspended nanowires grown across a ≈500 nm gap using a dwell time per point of 100 ms a) without laser treatment or a reactive co-flow and b) with both a reactive gas co-flow and laser assist. 10-sided polygons constructed c) without laser-assist and d) with laser-assist using a 10 µs pulse width with a pulse frequency of 100 Hz. e) is a plot of the resistivity of suspended nanowires as a function of beam dwell time under different growth conditions (see legend). The white arrows in part b highlight the morphological transition from standard EBID-like (darker) to higher purity (bright).

The resistivity of patterned nanobridges was measured to correlate the standard EBID growth conditions as well as the observed Pt grain coarsening and carbon reduction/transformation for the LAEBID growth conditions. For reference, bulk platinum has a resistivity on the order of 11 µΩ·cm. As shown in Figure 3a deposits grown using the MeCpPt(IV)Me_3_ precursor have a microstructure that consists of nanocrystalline platinum grains embedded in an amorphous carbon matrix which is consistent with many previous reports [61,71–72]. The nanogranular nature leads to intergranular tunneling and resistivities tunable over orders of magnitudes, all much higher than bulk platinum [73]. Figure 4a and Figure 4b show SEM images of nanobridges grown with and without laser assist, respectively, and Figure 4e is a plot of the resultant calculated resistivity versus electron beam dwell time for various EBID conditions.

Purification of the nanobridges can be qualitatively indicated by their resistivity. Lower resistivity corresponds to a higher platinum concentration as well as graphitization of the amorphous carbon matrix. Figure 4 compares two nanobridges: a) grown under standard EBID and b) LAEBID with the laser driven at a frequency of 100 Hz with a 10 µs pulse width. Both bridges were grown using the same electron beam scanning parameters, namely a 4 second spot dwell to grow a short vertical pillar followed by a 100 ms dwell per nm for the segment spanning the gap, where each half of the bridge was grown in parallel with the other half by alternating the beam from side to side (see Supporting Information File 1, Figure S2 for more details). The SEM images show increased contrast on the bridge that was grown with the laser assist, especially near the center and higher-up on the pillars away from the substrate.

Figure 4e reveals several trends in the resistivity of the 3D EBID and laser-assisted EBID structures. First, for the standard EBID growths, the resistivity decreases almost 4 orders of magnitude with increasing dwell time over the range measured. This is consistent with previous work illustrating that the C/Pt ratio (and consequently the resistivity) decreases from nearly 8/1 to 5/1 with increasing current [71]. HAADF STEM images of the standard EBID segments grown with different dwell time are shown in Supporting Information File 1, Figure S5. Secondly, the laser-assisted EBID growths also follow a similar trend, albeit a narrower range, in that the resistivity decreases as a function of increasing dwell time which is consistent with the TEM observations. Thirdly, and as-expected, the resistivity for the laser-assisted EBID reduces approximately two orders of magnitude relative to the standard EBID conditions. Finally, the effect of the O_2_ co-flow is negligible for both the standard EBID and LAEBID which is consistent with the fact that the observed segment angle versus dwell time plots were not affected. It is worth noting that the deposit resistivity is in actuality likely lower than reported here as the measurements includes a possibly significant contribution from contact resistance and the unpurified carbon-rich material near the substrate surface. However, the relative changes in resistivity based on different fabrication processes remain valid, indicating significant improvement with the use of in situ laser treatment.

One of the side effects of purification is necessarily the volume reduction associated with the impurity removal from the deposit. This volumetric reduction can be detrimental to the fidelity of complex shapes when purified ex situ – especially complex shapes that contain many vertices [74] (see Supporting Information File 1 for images). Figure 4c and Figure 4d show 10-sided polygons grown without and with laser-assist, respectively, which illustrates that the in situ laser assisted process can preserve the shape fidelity with higher resolution due to isotropic volumetric reduction.

### Thermal simulations

Thermal simulations were performed in order to approximate the thermal profile of the laser exposure during deposition and its impact on the resultant grain structure and carbon removal. In standard EBID using the MeCpPt(IV)Me_3_ precursor, the initial reaction leading to condensation occurs with the cleavage of a single bond between a methyl group and the platinum atom, surmised to occur primarily through dissociative electron attachment (DEA) along with smaller contributions from other reaction pathways [44]. After this initial cleavage event, subsequent electron exposure enables further decomposition of the precursor, ultimately allowing the removal of more methyl groups, though still falling short of complete removal.

There is a different situation at elevated temperatures. MeCpPt(IV)Me_3_ was originally developed for use as a thermal chemical vapor deposition (CVD) precursor [75]; the thermal decomposition temperature on the order of 120 °C in the presence of H_2_ results in pure Pt films. Thus, with LAEBID we leverage the pulsed thermal energy of the laser to aid the removal carbon impurities from the EBID platinum where the process is likely facilitated by residual water vapor in the chamber [56,76].

As is shown in Figure 3b–d there appears to be enhanced coarsening of the grains with increasing dwell time. Notably, these wires were grown in parallel, and thus all of the wires were exposed to the same number of laser pulses. If run in series (and separated beyond the laser spot size) the longer dwell time would experience more laser pulses. Initially, we suspected that wider nanopillars grown with longer dwell times per pixel would heat to a greater extent because they are optically thin, and wider pillars would experience more absorption. However, thermal simulations do not bear this out as larger radius simulations actually have slightly lower peak temperature, due to enhanced thermal transport to the substrate. Still, the length of the nanopillars changes with increasing dwell time, which effectively thermally isolates the pillar from the substrate enabling an increase in temperature (see Supporting Information File 1, Figure S3 and Figure S6 for more information). We thus attribute the enhanced coarsening at higher dwell times to the increase in temperature from the pulsed laser during deposition.

**Figure 5:**
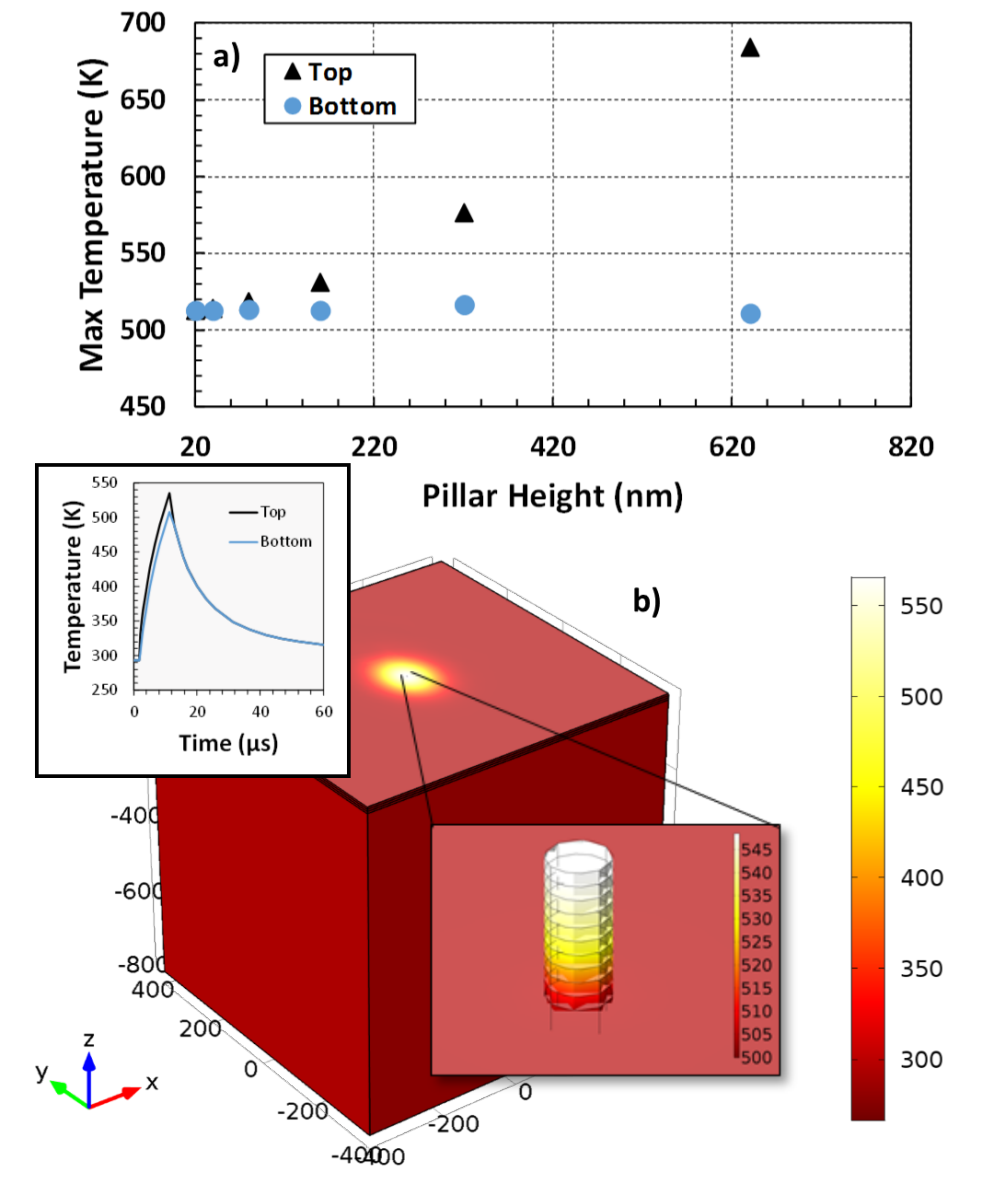
COMSOL™ simulation results showing the preferential heating at of the EBID deposit. a) is a plot of the maximum temperatures of both the top and bottom of the pillar at varying growth heights. The radius for the pillars was kept constant at 50 nm. b) shows the spatially resolved simulated temperatures in the system. Reading from right to left, the insets show a representative time-temperature plot for a single pillar grown to a height of 200 nm and a zoom-in on the pillar.

Visual inspection of the scanning electron micrograph in Figure 4b reveals two regimes for the LAEBID bridge; one with lower and one with higher SE yields. Indeed, STEM imaging corroborates that the bright areas correspond to higher purity and the lower SE yield corresponds to microstructure more closely related to as-deposited morphology. The variation in composition is caused by changes in the temperature profile within the segment as segment length increases: thermal transport to the substrate is limited by the pseudo 1D geometry of the segment. Figure 5a plots the simulated temperature at the top of an EBID Pt–C pillar and the substrate immediately beneath the base of the pillar (See Supporting Information File 1, Figure S3 and Figure S4 for more simulation details). The radius of the pillar is 50 nm and the pillar height varies from 20 to 640 nm. The experimental laser conditions are estimated to heat the silicon substrate to ≈510 K. As the aspect ratio of the pillar increases, the temperature at the top of the pillar increases, whereas the silicon substrate remains constant. Experimentally, when the pillar grows to roughly 250 nm, the temperature from the laser reaches a point at which significant purification is evident. According to simulation, this height corresponds to a temperature of ≈550 K.

## Conclusion

High fidelity 3D structures were fabricated by employing pulsed laser-assisted electron beam induced deposition with MeCpPt(IV)Me_3_ as a precursor gas. We investigated the internal morphology of freestanding structures grown using both standard EBID and LAEBID with STEM and EELS. Standard 3D EBID structures exhibit a grain size and resistivity dependence on electron beam dwell time. In the laser-treated deposits, Pt grain coarsening and a decrease in resistivity are observed at higher dwell times/higher segment angles. The 3D nanostructures maintain high fidelity during laser irradiation and realize a 100-fold decrease in resistivity when compared to standard EBID. This improvement in electron transport is attributed to carbon removal, graphitization, and Pt grain growth – all of which occur as a result of thermal energy from the pulsed laser system. Thermal simulations corroborate experimental data, showing that a temperature of >280 °C is required to catalyze the purification process. Importantly, this work was done in the context of maintaining a high degree of spatial control when constructing complex 3D mesh geometries. To this end, we demonstrated a method for calibrating the relationship between vertical and lateral growth. This method can be used in virtually any EBID system for a variety of different growth conditions.

## Supporting Information

File 1 3D Nanoprinting via LAEBID supplement.
This supplement describes the details of the shape fidelity of the in situ anneal, the bridge patterning strategy, the thermal simulation methods and variable, the STEM images of as-deposited wires, and the elemental mapping of the nanowires.
