# Supplementary material for: 3D Nanoprinting via laser-assisted electron beam induced deposition: growth kinetics, enhanced purity, and electrical resistivity
Source: Beilstein J Nanotechnol. 2017 Apr 7;8:801–12. doi: 10.3762/bjnano.8.83 (PMC5389181; doi:10.3762/bjnano.8.83)
Supplement: File 1 — 3D Nanoprinting via LAEBID supplement. This supplement describes the details of the shape fidelity of the in situ anneal, the bridge patterning strategy, the thermal simulation methods and variable, the STEM images of as-deposited wires, and the elemental mapping of the nanowires. [file Beilstein_J_Nanotechnol-08-801-s001.pdf]

Supporting Information

for

3D Nanoprinting via laser-assisted electron beam induced deposition: growth kinetics, enhanced purity, and electrical resistivity

Brett B. Lewis[1], Robert Winkler[2], Xiahan Sang[3], Pushpa R. Pudasaini[1], Michael G. Stanford[1], Harald Plank[2,4], Raymond R. Unocic[3], Jason D. Fowlkes[1,3] and Philip D. Rack[*1,3]

Address: [1]Materials Science and Engineering Department, University of Tennessee, Knoxville, TN 37996, USA, [2]Graz Centre for Electron Microscopy, Steyrergasse 17, 8010 Graz, Austria, [3]Center for Nanophase Materials Sciences, Oak Ridge National Laboratory, Oak Ridge, TN 37381, USA and [4]Institute of Electron Microscopy and Nanoanalysis, Graz University of Technology, Steyrergasse 17, 8010 Graz, Austria

Email: Philip D. Rack - prack@utk.edu
[*] Corresponding author

3D Nanoprinting via LAEBID supplement

Fidelity

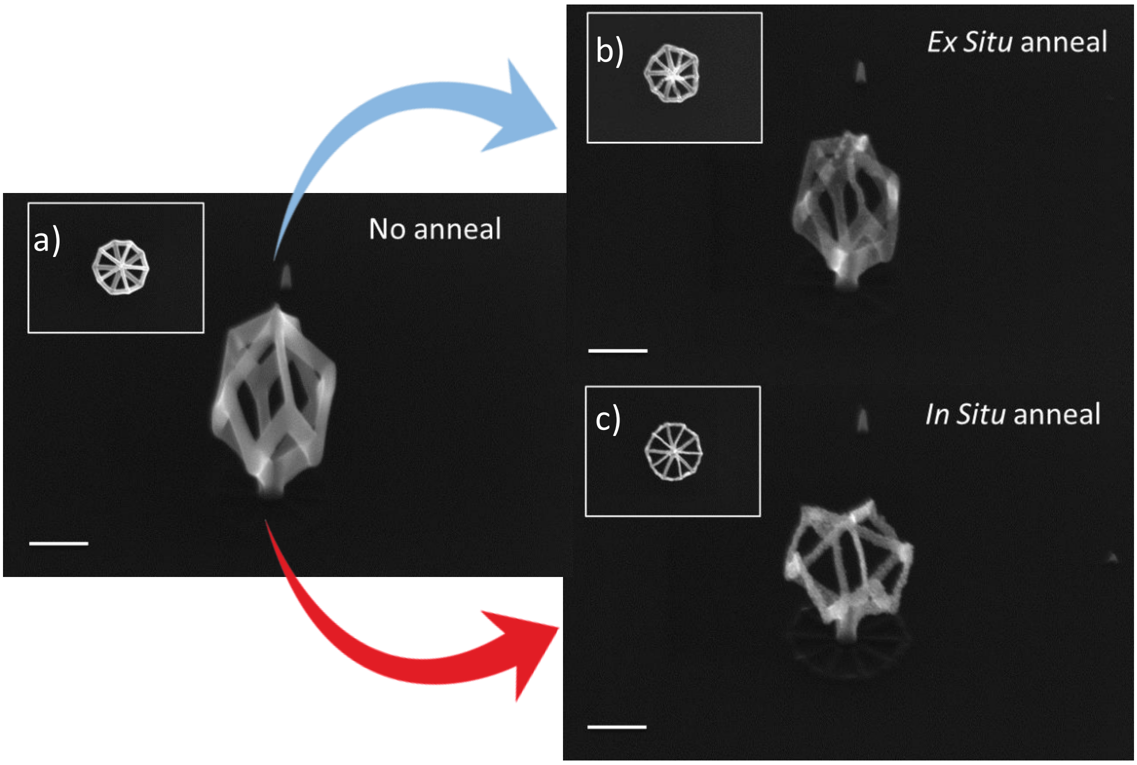

Figure S1: Cross-sectional SEM images demonstrating the utility of in situ laser purification compared to ex situ laser purification. a) shows a complex 10-sided polygon as-deposited without any laser annealing. b) shows the same polygon purified with laser annealing ex situ and c) shows the same polygon purified in situ. The insets show the top down SEM images corresponding to each cross-sectional image. This illustrates the importance of in situ purification strategies when dealing with complex 3D structures. The deformation is much larger with ex situ annealing than the in situ annealing. The scale bar is 100 nm.

Bridge Patterning

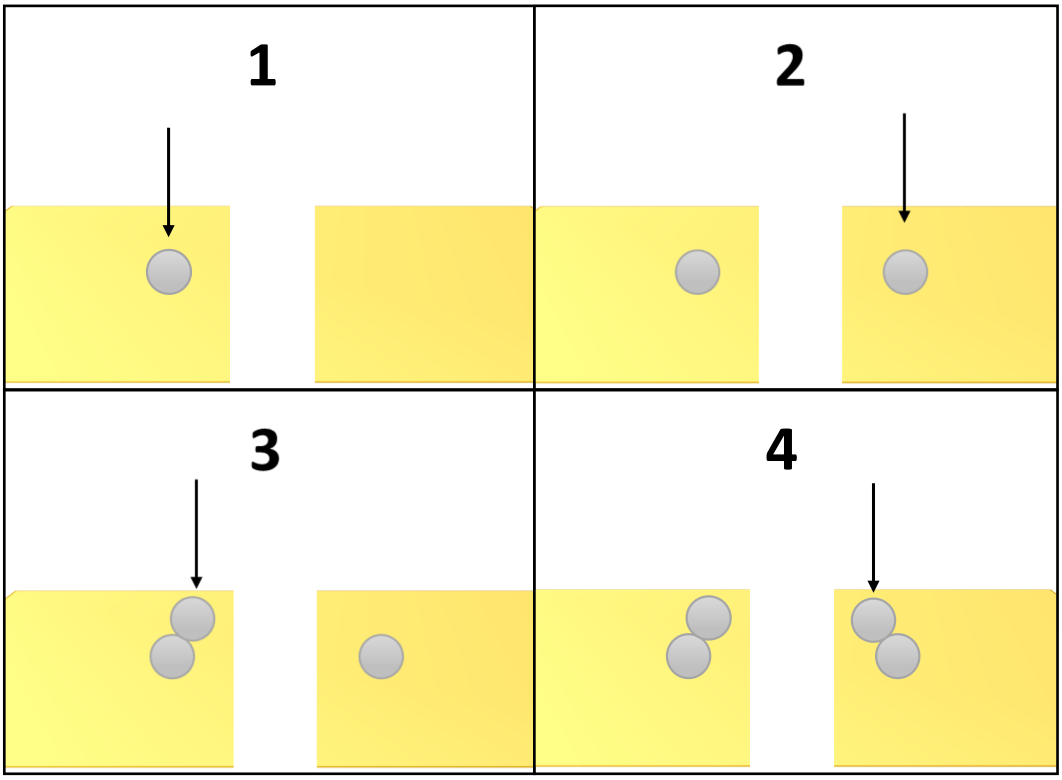

Figure S2: Schematic illustrating the alternating pattern of the nanobridges spanning the gap in the two-point probe contact pads. The arrow represents the electron beam location and the silver circles represent a deposit created during the beam dwell at the particular point. The patterning of the beam progresses from 1 to 4 and beyond bridging the gap by an alternating patterning strategy [1].

Temperature Simulations

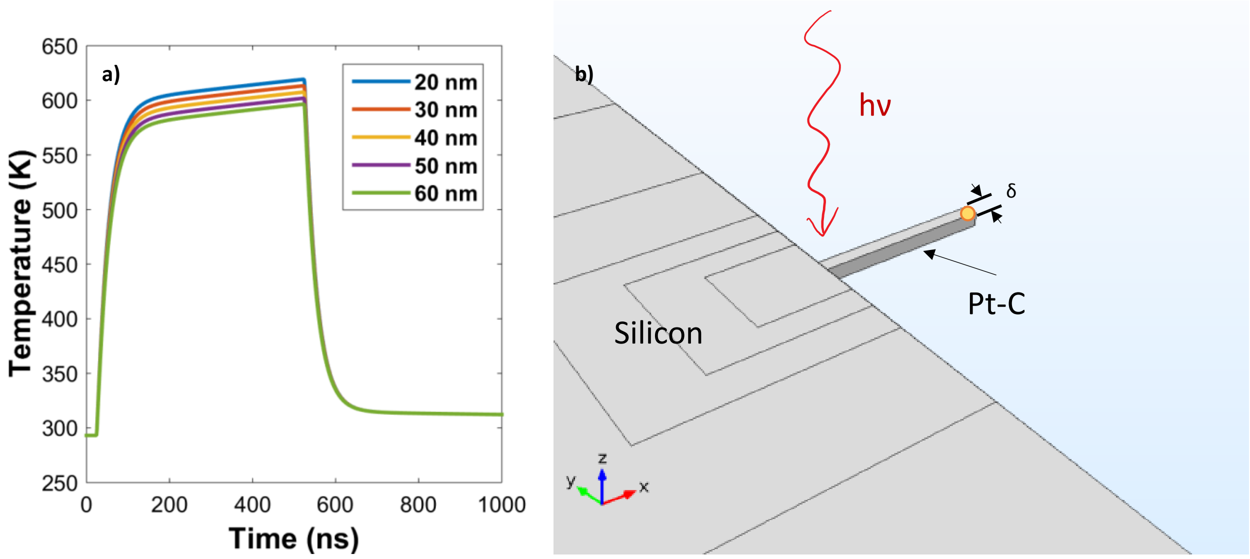

Figure S3: a) Time-Temperature simulation results of a nanopillar segment with constant length (600 nm) and various segment thicknesses (δ) as denoted in the legend. The pillar is placed at the edge of a solid silicon block directly horizontal from the substrate and perpendicular to the direction of the laser. The peak temperature slightly decreases as the thickness of the pillar grows due to enhanced coupling with the substrate. The temperature profiles are taken from the top of the pillar; the farthest point away from the substrate, illustrated by an orange dot in the schematic. b) A schematic illustrating the simulation geometry.

COMSOL™ Multiphysics 5.0, a commercial finite element method software package, was used to simulate the thermal profile of a single laser pulse on the different geometries. The expression for heat delivered to the substrate from the laser is derived from the Beer-Lambert law:

$$Q_{in}(x,y,z) = Q_0(1-R_c) \times \frac{A_c}{\pi\sigma_x\sigma_y} \times G(x,y) \times f(t) \times \exp(-A_c z)\ ;$$

where Q_0 is the optical power of the laser, R_c is the reflectivity, A_c is the absorption coefficient, $G(x,y)$ is the 2-dimensional Gaussian laser profile, σ_x and σ_y are the $1/e$ radii of the Gaussian laser profile, $f(t)$ is the laser on time, and z is the depth from the surface. The reflectivity and absorption coefficient are given by:

$$R_c = \frac{(n-1)^2+k^2}{(n+1)^2+k^2};$$

$$A_c = \frac{4\pi k}{\lambda};$$

where the values for n, k, and lambda are given in Table S1 for both silicon and EBID platinum. The laser on time, $f(t)$, was approximated as a rectangular pulse with a 10 ns ramp time and a pulse width of 10 µs. The following time dependent heat equation was used to simulate the heat transfer throughout the silicon substrate:

$$\rho C_p \frac{\partial T}{\partial t} + \rho C_p \boldsymbol{u} \cdot \nabla T = \nabla \cdot (\kappa \nabla T) + Q_{in}(x, y, z);$$

where ρ is the material density, C_p is the heat capacity at constant pressure, $\boldsymbol{u}$ is the velocity vector for thermal transport (which for a stationary system such as this is equal to 0), and κ is the thermal conductivity. Convective heat transfer to the surrounding atmosphere was neglected since irradiation conditions were under high vacuum. T_0 was defined as 293.15 K. A backward differentiation formula time-stepping method with strict time steps of 10 ns was used to generate the temporal temperature evolution for system during laser irradiation. The asymmetry of the Gaussian beam profile accounts for the angle of the laser with respect to the substrate during pillar growth (52°). For more information concerning the EBID platinum material values consult [2].

The material values used in Table S1 including the optical constants, density, heat capacity, and thermal conductivity are held constant for the sake of simplicity in this model. In reality, these values change throughout the course of the laser pulse as a function of both temperature and morphological evolution.

In reference to the silicon substrate, the absorption coefficient will change with temperature because the extinction coefficient is temperature dependent. In general, *k* increases slightly with temperature whereas *n* stays relatively stable [3] leading to a larger absorption coefficient and therefore a hotter substrate temperature. The heat capacity at constant pressure will also change with temperature increasing slightly (~14%) from room temperature to 600 K [4]. This increase will effectively decrease the temperature of the irradiated silicon. The thermal conductivity of silicon scales as $T^{-1.2}$ [5] meaning that over the simulated temperature ranges, the thermal conductivity will decrease by roughly a factor of 2. In our described system this decrease in thermal conductivity would lead to an increase in overall temperature of the silicon substrate.

With regards to the platinum deposit, the dominant mechanism for change is the granular Pt-amorphous carbon composite to metallic transition. As carbonaceous material is removed, the density and thermal conductivity will increase and the *n* and *k* values will increase as well. The thermal conductivity of the as-deposited PtC_x is largely dominated by the thermal conductivity of the amorphous carbon, which is very low. As carbon is removed and graphitized, the thermal conductivity of the wire is expected to increase which will decrease the temperature of the wire moderately self-limiting the heating from the laser pulse. As the wire becomes more metallic, the corresponding increase in *n* and *k* values will increase both the reflectivity and absorption coefficient. It is expected that the temperature of the wire would experience a relative increase. This prediction arises from the observation that PtC_x has a penetration depth longer than the dimensions of the wire itself (~234 nm). Platinum, on the other hand, has an absorption depth of ~8 nm at room temperature. This means that the absorption depth will shrink as the carbon is removed leading to more of the incident laser irradiation being absorbed.

Table S1: *Simulation parameters.*

SYMBOL	NAME	VALUE SILICON	VALUE PtC_X
**n**	Real index of refraction	3.63	1.87
**k**	Imaginary index of refraction	0.0023	0.311
**λ**	Laser wavelength	915 nm	915 nm
**ρ**	Material Density	2.329 g/cm^3	4.550 g/cm^3
C_p	Heat capacity at constant Pressure	700 J/(kg·K)	360 J/(kg·K)
**κ**	Thermal Conductivity	130 W/(m·K)	8.6 W/(m·K)
Q_0	Optical Laser Power	20 W	20 W
σ_x	1/*e* radius in the *x* direction	50 µm	50 µm
σ_y	1/*e* radius in the *y* direction	35.35 µm	35.35 µm
R_p	Pillar Radius	n/a	50 nm

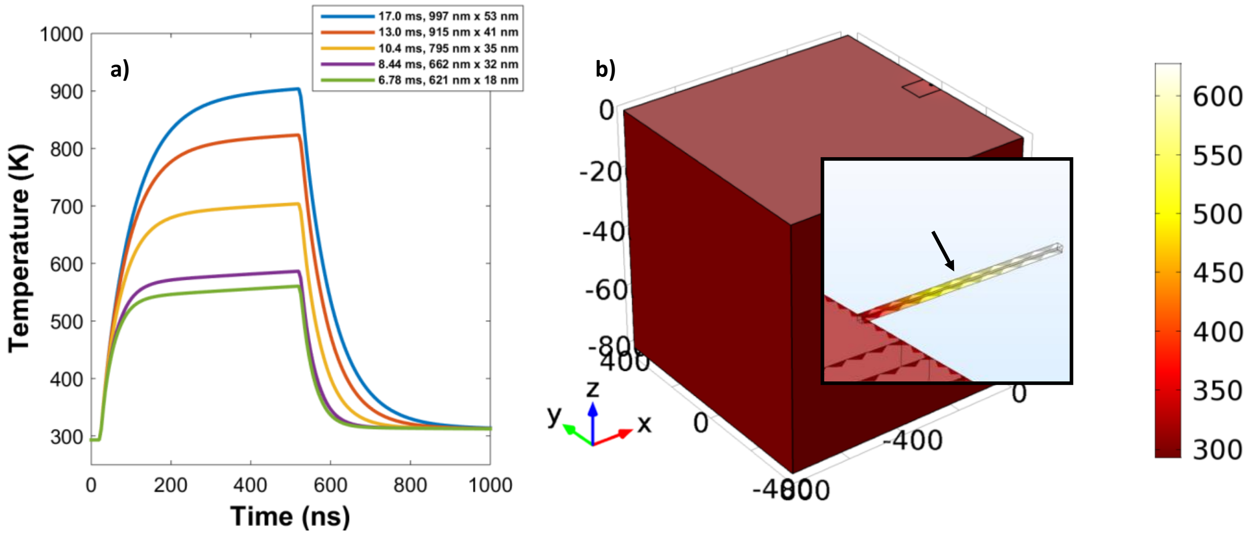

Figure S4: a) Time-temperature simulation results a nanopillar segments grown at the various lengths and widths. These lengths correspond to the experimental values of the pillars grown and analyzed using STEM and EELS. The values in the legend are of the following format: dwell time, length *x* diameter. The temperature was sampled at the center of the segment to correspond with the STEM images shown in Figure 3 of the article. The maximum values for the temperature show that the segment attains a higher heat for the longer segments. This confirms the observation that higher temperatures produce purer structures with larger Pt grain sizes. b) Spatially resolved temperature profile of the smallest pillar corresponding to the green curve in part a. The inset is a zoom-in of the pillar and the arrow depicts the point from which the temperature profiles are sample from in part a.

STEM Images

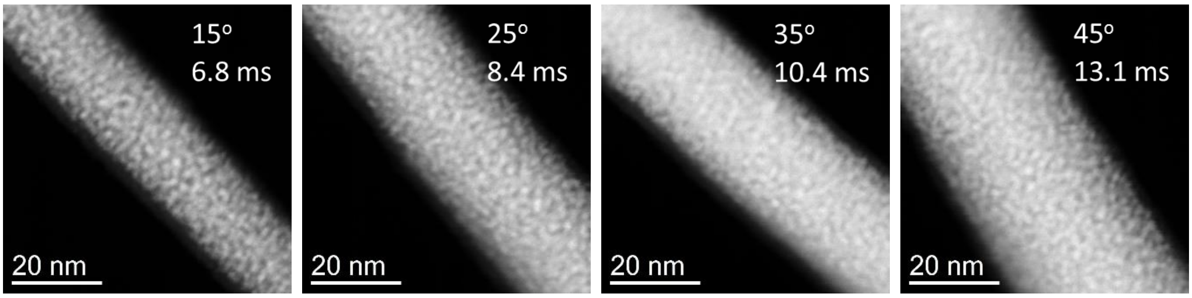

Figure S5: High angle annular dark field (HAADF) STEM images of as deposited segments showing the grain size coarsening as a function of increasing dwell time.

Elemental Mapping

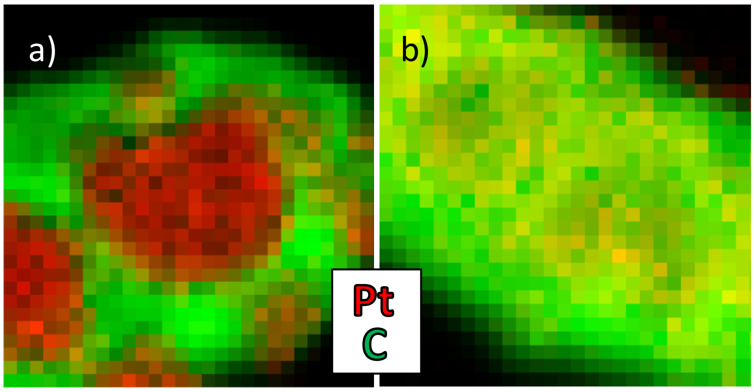

Figure S6: STEM EELS maps showing the distribution of Pt (red) and C (green) in laser treated EBID segments. a) Near the tip (far from the substrate surface) of a 55°, 17.0 ms segment grown off a silicon wafer edge. b) Near the bottom (closer to the substrate surface) of a 55°, 17.0 ms segment grown off the silicon wafer edge. The compositional difference highlights significant effect thermal transport has on the extent of purification.

Reference

1. Winkler, R.; Schmidt, F.-P.; Haselmann, U.; Fowlkes, J. D.; Lewis, B. B.; Kothleitner, G.; Rack, P. D.; Plank, H., *ACS Applied Materials & Interfaces* **2017**, *9*, 8233–8240.

2. Stanford, M. G.; Lewis, B. B.; Noh, J. H.; Fowlkes, J. D.; Roberts, N. A.; Plank, H.; Rack, P. D., *ACS Appl Mater Interfaces* **2014,** *6* (23), 21256-21263.

3. Weakliem, H. A.; Redfield, D., *Journal of Applied Physics* **1979,** *50* (3), 1491-1493.

4. Okhotin, A.; Pushkarskii, A.; Gorbachev, V., *Thermophysical Properties of Semiconductors*. Atom Publishing House: Moscow, 1972.

5. Glassbrenner, C. J.; Slack, G. A., *Physical Review* **1964,** *134* (4A), A1058-A1069.
